# Association of hemoglobin glycation index and its interaction with obesity/family history of hypertension on hypertension risk: a community-based cross-sectional survey

**DOI:** 10.1186/s12872-020-01762-0

**Published:** 2020-11-04

**Authors:** Jing Mi, Jian Song, Yingying Zhao, Xuesen Wu

**Affiliations:** 1 School of Public Health, Bengbu Medical College, 2600 Donghai road, Bengbu, 233000 Anhui Province China; 2 Bengbu Health Board, 568 Nanhu road, Bengbu, 233000 Anhui Province China

**Keywords:** Hypertension, Hemoglobin glycation index, Interaction

## Abstract

**Background:**

Hemoglobin glycation index (HGI) is considered to be a convenient measurable indicator to assess the inter-individual variation of HbA1c. In the present study, we tested the relationship between HGI and risk of hypertension, and further explored the possible interacting influences of HGI with other such factors on hypertension risk among Chinese individuals.

**Methods:**

The eligible subjects were chosen from a community-based cross-sectional survey in China. We collected relevant data and clinical indicators for each participant. HGI was calculated as “measured HbA1c-predicted HbA1c” and divided into four categories according to quartile. The following indicators were used to assess interactive effects: (1) relative excess risk due to interaction (RERI); (2) attributable proportion due to interaction (AP); and (3) synergy index (SI). Statistical analysis was performed using R software.

**Results:**

Specifically, 1777 eligible participants were selected in this cross-sectional survey. There were 433 subjects who were identified to have hypertension (24.4%). A significant increase in the prevalence of hypertension from Q1 to Q4 of HGI was observed (*p* < 0.001). Multivariable logistic model demonstrated that subjects at the highest HGI group had a substantially increased risk of being hypertensive than subjects in the first quartile of HGI, as indicated by the OR value of 1.87 (95% CI 1.26–2.78). Moreover, a significant interaction between family history of hypertension and HGI on hypertension risk was detected (RERI: 1.36, 95% CI 0.11–2.63; AP: 0.43, 95% CI 0.17–0.69; and SI:2.68, 95% CI 1.10–6.48). The interactive effect between HGI and abdominal obesity was also found to be significant, as estimated by the value of RERI (1.04, 95% CI 0.24–1.85), AP (0.33, 95% CI 0.11–0.56) and SI (1.96, 95% CI 1.01–3.79). However, in the analysis of the interaction between HGI and general obesity, only the AP value (0.28, 95% CI 0.01–0.54) was observed to be significant.

**Conclusion:**

High HGI was independently associated with the risk of hypertension. Moreover, HGI significantly shared interactions with obesity and family history of hypertension that influenced the risk of hypertension.

## Introduction

Hypertension, along with complications such as stroke and chronic kidney disease, substantially consumes social and medical resources while heavily burdening families and society [1]. Nearly 43% of cardiovascular events were found to be attributed to hypertension [2]. Meanwhile, a number of national surveys have showed that the prevalence of hypertension is generally increasing in China [3–5]. Therefore, it is a matter of considerable importance to understand its pathogenesis in reducing cardiovascular disease mortality and disease burden.

Individuals with hypertension are often accompanied by abnormal glucose metabolism [6]. Glycated hemoglobin (HbA1c), an indicator of blood glucose stability, is extensively used in modern clinical practice and epidemiological surveys [7]. However, evidence suggests that HbA1c levels are affected by average glycemia as well as the biological differences between individuals [8]. Accordingly, in individuals with similarly average blood glucose levels, HbA1c values may appear to be consistently higher or lower than others [8, 9]. Therefore, researchers introduced the hemoglobin glycation index (HGI) as an indicator to assess the inter-individual variation of HbA1c, which could reflect individuals’ glycation tendency [10]. HGI was defined as the discrepancy between the measured HbA1c and predicted HbA1c based on plasma glucose levels [10]. Enhanced HGI levels represented a higher sensitivity to protein glycosylation and increased accumulation of advanced glycation end products (AGEs) [11]. In diabetic subjects, HGI was reported to be independently associated with a higher risk of diabetic complications like diabetic retinopathy and nephropathy [12]. For every standard increase in HGI, the risk of diabetic microvascular complications would increase by 14% [12]. Moreover, in nondiabetic individuals, a significantly positive relationship between HGI and cardiometabolic risk factors clustering was observed [13]. However, thus far, a limited number of articles have evaluated the relationship between HGI and hypertension risk. Additionally, hypertension is a complicated disease stemming from multiple factors, and several studies have investigated the combined impacts between such factors on hypertension risk. For instance, obesity was reported to significantly interact with the family history of hypertension, increasing the risk of hypertension [14]. Insulin resistance had a combined effect with obesity and family history of hypertension that substantially enhanced the risk of hypertension [15]. However, whether HGI has an interactive effect with other influencing factors on hypertension risk is unknown.

To gain a deeper understanding of the relationship between inter-individual variation of HbA1c and hypertension risk, by utilizing data from a community-based cross-sectional survey in China, this study aimed to:(1) analyze the independent association between HGI and hypertension risk; (2) explore possible interactive influences of HGI with other associated factors on hypertension risk.

## Methods

### Study population

This study was a community-based cross-sectional survey that was performed in Longzihu, Bengbu, China. Eligible residents were selected using stratified multi-stage cluster sampling method. All enrolled participants must reach all the below conditions: (1) a local resident in selected communities (continuous residence ≥ 6 months in the past year); (2) have the ability to complete the whole survey independently; (3) agreed to participate in this survey voluntarily and be willing to cooperate in completing the questionnaire and medical examination. Exclusion criteria included: (1) those with mental disorders or disturbances of consciousness due to various reasons that affected normal communication; (2) pregnant or lactating women; (3) individuals with incomplete survey. All respondents provided informed consent before data collection. The Ethics Committee of Bengbu Medical College reviewed this study.

### Data collection

1. Questionnaire survey: A self-designed questionnaire was utilized, and all investigators received uniform training. The questionnaire included general information such as gender, age, past medical history, income, marriage status, smoking, educational level, and family history of hypertension. Individuals with at least one parent or sibling with hypertension were defined as having a positive family history of hypertension. The questionnaire survey developed for this study is provided as Additional File 1.
2. Anthropometric measurements: During measurement, the subjects were required to stand, take off their shoes and hats, and wear lightweight clothing. Waist circumference (WC) was measuring using a standard soft ruler horizontally surrounding the abdomen placed at the midpoint of the line connecting the anterior superior iliac crest and the lower edge of the 12th rib. The BMI was defined as the weight (kg) divided by the height squared (m^2^). According to the Chinese Health Industry Standard of Adult Weight Determination (WS/T428-2013), individuals with BMI ≥ 28 kg/m^2^ were considered to have general obesity [16]. Males with WC ≥ 90 cm and females with WC ≥ 85 cm were considered to have abdominal obesity, respectively [16].
3. Blood pressure (BP) measurement: After the participants rested quietly for about 10 min, the investigators performed three consecutive measurements for each participant using the standard method [17]. Finally, the average of the three measurements was taken as the individual BP value. Those who met any of the below conditions were defined as hypertension:(1) under therapy with antihypertensive drugs; (2) Systolic BP (SBP) ≥ 140 mmHg; (3) Diastolic BP (DBP) ≥ 90 mmHg [18].
4. HGI calculation: Subjects fasted for at least 8 h, and 5 ml of cubital vein blood was collected from the morning of the day of the physical examination. Fasting plasma glucose (FPG), HbA1c, triglycerides (TG) and total cholesterol (TC) were examined. A linear regression model was established between HbA1c and FPG: predicted HbA1c = 0.256 × FPG (mmol/l) + 3.840 (r = 0.608, *p* < 0.001). The HGI was calculated as “measured HbA1c- predict HbA1c” [10], then divided into four categories according to quartile (Q1–Q4).

### Statistical methods

Statistical analysis was performed using R software. Quantitative data was described by their mean ± standard deviation or median (P25, P75), and their differences between HGI quartiles (Q1–Q4) were compared by analysis of variance or the Kruskal–Wallis H test. Categorical data was described by percentages (%) and their differences across HGI groups were compared using the Chi-square test. The independent association between HGI and hypertension risk was evaluated by univariate as well as multivariable logistic regression model. The adjusted variables included age, gender, income, marital status, educational level, smoking, family history of hypertension, obesity, FPG, HbA1c, TG and TC. Using the lowest HGI group (Q1) as reference, we calculated the OR (odds ratio) value and its corresponding 95%CI for the other HGI groups (Q2, Q3, and Q4). Since the interaction analysis required the variables to be dichotomous, a ROC (receiver operating characteristic) curve analysis was conducted to calculate the best threshold of HGI. Finally, the following interactive indicators were calculated respectively: (1) the relative excess risk due to interaction (RERI); (2) attributable proportion due to interaction (AP) and (3) the synergy index (SI) [19, 20]. Interaction was considered significant when the 95%CI of RERI, AP and SI did not comprise 0.0 and 1, separately [19, 20]. All statistical analyses were performed with α = 0.05 as the test level.

## Results

### General Characteristics of enrolled subjects

**Fig. 1:**
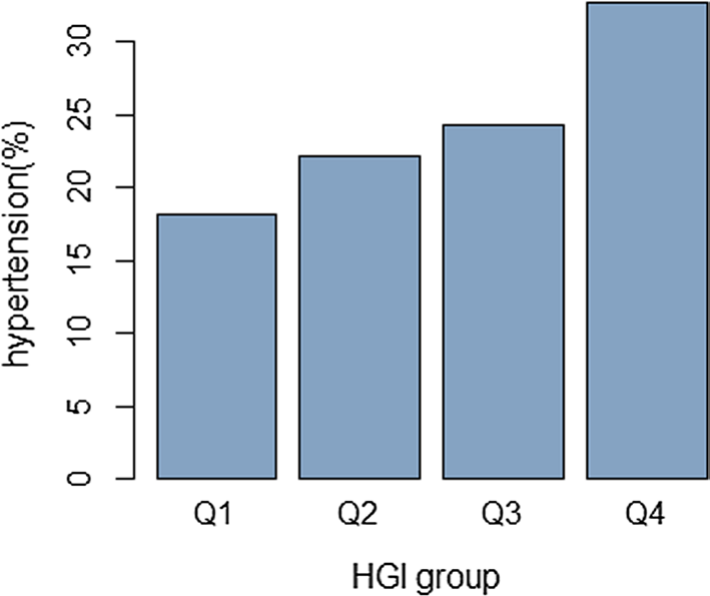
The prevalence of prehypertension across HGI quartiles (The prevalence of prehypertension significantly increased across HGI quartiles: P _for trend_ < 0.001)

In this cross-sectional survey, 1777 eligible participants were selected with a mean age was 60.82 ± 11.24 years. Among the respondents, 433 subjects (24.4%) were found to have hypertension. The comparisons of the general characteristics across HGI groups are listed in Table 1. There was a remarkable difference of age across HGI groups (*p* < 0.001), and the subjects among Q4 group were the oldest. The distribution of gender across the groups of HGI were found to be nonsignificant (*p* = 0.796). A remarkable difference of smoking rate was observed across HGI groups (*p* = 0.023). In terms of anthropometric features, we observed a remarkable difference in WC across the HGI groups (*p* < 0.001), however, BMI was not found to be significant (*p* = 0.610). From Q1 to Q4 of HGI, the level of HbA1c dramatically increased (*p* < 0.001). Also, significant differences in FPG (*p* < 0.001) as well as TG (*p* = 0.044) between the groups were observed. Moreover, a significant increase in the prevalence of hypertension from Q1 to Q4 of HGI was seen (*p* < 0.001), as shown in Fig. 1. However, no statistically significant differences were detected for the following variables: marital status (*p* = 0.178), family history of hypertension (*p* = 0.452), education level (*p* = 0.171), income (*p* = 0.539) and TC (*p* = 0.531).

**Table 1:** Basic characteristic of the study participants

	HGI				F/H/χ^2^	* p*
	Q1	Q2	Q3	Q1		
Age (years)	59.69 ± 11.38	59.32 ± 11.37	61.96 ± 11.25	62.30 ± 10.67	8.303^a^	< 0.001
Male (N (%))	192 (43.2)	184 (41.3)	180 (40.5)	192 (43.2)	1.022^b^	0.796
Marital status (N (%))					4.916^b^	0.178
Currently married	367 (82.7)	388 (87.2)	366 (82.4)	376 (84.7)		
Currently not married	77 (17.3)	57 (12.8)	78 (17.6)	68 (15.3)		
Educational level (N (%))					5.009^b^	0.171
Middle school or lower	323 (72.7)	294 (66.1)	312 (70.3)	304 (68.5)		
High school or higher	121 (27.3)	151 (33.9)	132 (29.7)	140 (31.5)		
Income (yuan) (N (%))					2.167^b^	0.539
< = 2000	248 (55.9)	246 (55.3)	242 (54.5)	228 (51.4)		
> 2000	196 (44.1)	199 (44.7)	202 (45.5)	216 (48.6)		
Family history of hypertension (N (%))	93 (20.9)	76 (17.1)	81 (18.3)	78 (18.5)	2.630^b^	0.452
Smoking (%)	114 (25.7)	124 (27.9)	134 (30.2)	154 (34.7)	9.504-	0.023
BMI (kg/m^2^)	24.56 (22.24,26.61)	24.28 (22.21,26.77)	24.62 (22.32,27.00)	24.46 (22.51,27.00)	1.823^c^	0.61
WC (cm)	85.00 (80.00,90.00)	85.00 (79.00,91.00)	86.00 (80.00,93.00)	87.00 (80.00,93.00)	17.539^c^	< 0.001
FPG (mmol/L)	5.86 ± 1.73	5.33 ± 1.52	5.23 ± 1.55	5.54 ± 2.01	11.639^a^	< 0.001
HbA1c (%)	4.20 (3.80,4.60)	4.70 (4.50,5.10)	5.30 (5.10,5.70)	6.50 (5.90,7.50)	1064.07^c^	< 0.001
TG (mmol/L)	1.36 (0.97,1.89)	1.35,0.96,1.99)	1.45 (0.95,2.00)	1.53 (1.02,2.15)	8.107^c^	0.044
TC (mmol/L)	4.92 (4.30,5.51)	4.98 (4.34,5.70)	4.96 (4.20,5.66)	5.00 (4.24.5.76)	2.206^c^	0.531
Hypertension (N (%))	81 (18.2)	99 (22.2)	108 (24.3)	145 (32.7)	26.680^b^	< 0.001

BMI: body mass index; WC: waist circumference; FPG: fasting plasma glucose; TG: triglycerides; TC: total cholesterol

^a^Analysis of variance;

^b^Chi-square test;

^c^Kruskal-Wallis H test

### Association between HGI and hypertension risk

The results of the univariate logistic regression analysis indicated that when compared to the reference group (Q1), individuals in Q3 as well as the highest group (Q4) had a significantly higher risk of acquiring hypertension (OR: 1.44, 95% CI 1.04–1.99 and OR: 2.17, 95% CI 1.59–2.97). After adjusting for confounding factors including HbA1c levels, individuals in the highest HGI group still remained a significantly increased risk of being hypertension than subjects at the first quartile of HGI, as indicated by the OR of 1.87 (95% CI 1.26–2.78). When treating HGI as a continuous variable, the corresponding OR (1.16, 95% CI 1.06–1.27) suggested that a per unit increase of HGI would confer a 1.16-fold risk of acquiring hypertension. These results are presented in Table 2.

**Table 2:** HGI and risk of hypertension by logistic regression analysis

Quartiles of HGI	N	Hypertension (%)	Unadjusted model	Multivariate model^a^	Continuous (per 1 SD)^a^
			OR (95%CI) P	OR (95%CI) P	OR (95%CI) P
Q1	444	18.2	Ref. -	Ref. -	1.16 (1.06–1.27) 0.001
Q2	445	22.2	1.28 (0.92–1.78) 0.138	1.33 (0.94–1.89) 0.098	
Q3	444	24.3	1.44 (1.04–1.99) 0.027	1.34 (0.94–1.91) 0.088	
Q4	444	32.7	2.17 (1.59–2.97) < 0.001	1.87 (1.26–2.78) < 0.001	

^a^Adjusted for age, gender, income, marital status, educational level, smoking, family history of hypertension, obesity, FPG, HbA1c, TG and TC

### Interaction between HGI and hypertension risk

Subjects were then classified into four subgroups based on their HGI level as well as their family history of hypertension, general obesity, and abdominal obesity, respectively, as presented in Table 3. Individuals with high-HGI and a positive family history of hypertension possessed the highest risk of getting hypertension as compared to those with low-HGI and a negative family history of hypertension (OR:3.18, 95% CI 2.05–4.94). Moreover, a significant interaction was detected between the HGI and family history of hypertension on hypertension in all indicators: RERI (1.36, 95% CI 0.11–2.63), AP (0.43, 95% CI 0.17–0.69) and SI (2.68, 95% CI 1.10–6.48). Compared to individuals with a low-HGI and BMI < 28, the corresponding adjusted ORs (95%CI) for subjects with low-HGI and BMI ≥ 28, high HGI and BMI < 28, high HGI and BMI ≥ 28 were 1.38 (0.96–1.99), 1.57 (1.05–2.34) and 2.70 (1.83–3.98), respectively. However, only the AP value was observed to be significant (0.28, 95% CI 0.01–0.54). Similarly, the hypertension risk among subjects with a high-HGI accompanied by abdominal obesity was 3.14 times that of those in the reference group. Moreover, the interactive effect between HGI and abdominal obesity was determined to be significant, as shown by the values of RERI (1.04, 95% CI 0.24–1.85), AP (0.33, 95% CI 0.11–0.56) and SI (1.96, 95% CI 1.01–3.79).

**Table 3:** The interactive effects of HGI with obesity, family history of hypertension on risk of hypertension

Variables		OR^2^(95%CI)	Measures of interaction^b^		
			RERI	AP	SI
HGI^a^	Family history of hypertension		1.36 (0.11–2.63)^c^	0.43 (0.17–0.69)^c^	2.68 (1.10–6.48)^c^
Low	No	1 (ref)			
Low	Yes	1.32 (0.90–1.93)			
High	No	1.50 (1.08–2.09)			
High	Yes	3.18 (2.05–4.94)			
HGI^a^	General obesity		0.75 (− 0.05–1.54)^d^	0.28 (0.01–0.54)^c^	1.78 (0.85–3.74)^d^
Low	No	1 (ref)			
Low	Yes	1.38 (0.96–1.99)			
High	No	1.57 (1.05–2.34)			
High	Yes	2.70 (1.83–3.98)			
HGI^a^	Abdominal obesity		1.04 (0.24–1.85)^c^	0.33 (0.11–0.56)^c^	1.96 (1.01–3.79)^c^
Low	No	1 (ref)			
Low	Yes	1.39 (1.01–1.92)			
High	No	1.71 (1.19–2.45)			
High	Yes	3.14 (2.30–4.28)			

^a^Grouped by cutoff value (0.06) based on ROC curve analysis;

^b^Adjusted for age, gender, income, marital status, educational level, smoking, family history of hypertension, obesity, FPG, HbA1c, TG and TC

^c^*p* < 0.05;

^d^*p* > 0.05;

## Discussion

HGI, an indicator used to assess the inter-individual variation of HbA1c, reflects the difference in the degree of hemoglobin glycosylation at a given plasma glucose level [10]. In addition to being influenced by blood glucose concentrations, the individual differences of HbA1c may be also associated with biological factors that influence non-enzymatic protein glycation such as genetics, and the life cycle of red blood cells [21].Various studies have confirmed that there are consistent inconsistencies between HbA1c and other clinically used blood glucose homeostasis indicators, such as fructosamine and mean blood glucose [8, 21, 22]. An increasing amount of evidence shows that such inconsistencies may affect the accuracy of HbA1c in management of diabetes and its other applications. For instance, the Action to Control Cardiovascular Risk in Diabetes (ACCORD) trial stated that interventions aimed solely at reducing HbA1c levels in diabetic patients did not reduce the incidence of cardiovascular events. Conversely, the risk of mortality increased in the intensive therapy group (target HbA1c < 6%) compared to the standard treatment group (target HbA1c 7.0% to 7.9%) [23]. However, the hazards or benefits from intensive glycemic control could be identified by HGI subgroups, suggesting that HGI should also be considered in addition to HbA1c levels [24]. Therefore, some researchers proposed that HGI may serve as an alternative marker for diseases management and prediction.

This study found that HGI serves as an independent risk factor in the onset of hypertension, regardless of HbA1c levels. Similarly, HGI was reported to be positively correlated with cardo-metabolic risk factors, independent of other glucose indexes, including HbA1c and post-load glucose concentrations [25]. A ten-year prospective cohort survey in Korean demonstrated that baseline HGI was significantly associated with the incidence of cardiovascular disease even after controlling HbA1c levels [26]. Overall, this strongly suggested that HGI may confer additional influences on cardiovascular diseases over other glucose measurements. In addition, this study used FPG rather than mean blood glucose to calculate the HGI for several reasons. First, it was indicated that HGI calculated by average total glucose had significantly high correlation with that by prebreakfast FPG only [27]. Second, FPG is comparatively more common and easily available in daily practice, whereas mean blood glucose data is not easily accessible and inconvenient to ascertain. Furthermore, a number of past studies have used FPG to calculate HGI, and demonstrated that it was an efficient and powerful indicator reflecting variation of HbA1c and may be used to predict related outcomes of diabetes [25, 26].

The pathophysiological mechanisms that increase the HGI leads to an increased risk of hypertension, which are currently not fully understood. Enhanced HGI levels were more highly sensitivity to protein glycosylation and had increased accumulation of AGEs [28, 29]. The concentration of AGEs taken from measuring skin intrinsic fluorescence was found to be significantly increased with the increase in HGI, suggesting that subjects with high HGI levels may have higher levels of AGEs than that of other populations [30]. AGEs are intermediate products that respond to chronic hyperglycemia, which can alternate arterial stiffness and cause endothelial injury either directly or by binding to specific receptors to recognize AGEs modified proteins [31, 32]. Compared to normotensive subjects, individuals suffering from hypertension had a significantly higher concentration of plasma AGEs [33]. In spontaneously hypertensive rats, the levels of AGEs were shown to be elevated [34]. Meanwhile, AGEs accumulation was shown to be closely related with night-systolic blood pressure, subclinical vascular atheromatosis and expected 10-year cardiovascular death risk in subjects with successful renal transplant [35]. In addition, chronic inflammation was recognized to be involved in the development of hypertension [36]. The National Health and Nutrition Examination Survey previously suggested that HGI was independently associated with inflammatory biomarkers including C-reactive protein (CRP), polymorphonuclear leukocytes, and monocytes [37]. Following 6 weeks of low-AGEs diet intervention in diabetics, the concentrations of inflammatory markers such as high-sensitivity C-reactive protein and TNFα were significantly reduced [38]. Meanwhile, serum AGE levels were reported to be significantly independent with the homeostasis model assessment of insulin resistance (HOMA-IR) index, suggesting that AGEs may trigger various pathologies via IR [39]. Individuals with a high HGI displayed a higher degree of IR than those with a low HGI [25]. An investigation using an animal model demonstrated that oral intake of AGEs impaired insulin uptake and induced insulin resistance by altering insulin receptor signal transduction [40].

The present study demonstrated that a remarkable interaction exists between HGI and family history of hypertension in regard to hypertension risk. Hypertension is the result of a combination of genetic and environmental factors, and family history of hypertension serves as an important marker for genetic factors, which is often used as a proxy indicator to analyze the association between genetic factors and hypertension. Numerous studies have indicated that having family history of hypertension is a considerable risk factor of hypertension. After analyzing the interactions between HGI and obesity on hypertension, all interactive indicators remained significant between HGI and abdominal obesity, however, only one index was significant in terms of HGI and general obesity. BMI may rapidly and easily assess the overall degree of obesity, but WC rather than BMI better reflects the accumulation of abdominal fat [41]. Relevant studies have strongly suggested that abdominal obesity has a more substantial impact on cardiovascular diseases risk compared to general obesity. A seven-year cohort survey indicated that WC possessed a higher predictability in hypertension risk than BMI [42]. Here, we found that WC, instead of BMI, was found to be significantly increased across HGI groups, suggesting that HGI and WC are closely related. The interaction of HGI and obesity may increase the occurrence of hypertension through co-owned mechanisms, such as inflammatory responses and insulin resistance [31, 43]. Future research should further explore these interactive mechanisms, which may further elucidate the cause of hypertension.

Recently, several studies have been performed to analyze the practical applicability of HGI measurements. A cross-sectional study demonstrated that high HGI levels significantly increased the risk of coronary artery disease, stroke, and peripheral artery disease in individuals with an impaired glucose metabolism [44]. The Kangbuk Samsung Health Study in Korean indicated that individuals with the highest HGI levels had a 1.722-fold risk of incident coronary artery calcium compared to the bottom group regardless of HbA1c levels [45]. In type 2 diabetes HGI was proposed to be closely related with the severity of coronary heart disease, which contributed to cardiovascular risk stratification [46]. In terms of ischemic stroke patients with type 2 diabetes, HGI was suggested to be an independent poor prognostic factor [47]. Moreover, among nondiabetic individuals, a higher HGI was reported to be inde50pendently related with nonalcoholic fatty liver disease, chronic kidney disease, hepatic steatosis, and carotid intima-media thickness [25, 48–50]. Overall, substantial evidence has demonstrated the practical value of HGI in disease prediction and management.

The present study possesses some limitations. First, we cannot verify causality as this is a cross-sectional study, and prospective cohort studies should be done for further validation. Second, FPG and HbA1c were only measured once, however, it is common in epidemiological surveys. The day-to-day variations in blood glucose indicators levels were not considered. Third, the results did not generalize other ethnic groups as there were obvious ethnic differences in HbA1c levels [51, 52]. Fourth, some other variables such as mental stress and daily salt intake were not collected, which might influence the associations observed in present study.

## Conclusion

In conclusion, high HGI was independently associated with the risk of hypertension. Moreover, HGI significantly shared interactions with obesity and family history of hypertension that influenced the risk of hypertension. Since HGI is a conveniently obtainable indicator reflecting inter-individual variation of HbA1c, it may be extensively used in practice for a more personalized and comprehensive evaluation of hypertension risk. Meanwhile, exploring the processes that cause biological variation of HbA1c levels may introduce effective novel strategies in the early prevention of hypertension and low blood pressure.

## Data Availability

The datasets used and/or analyzed during the current study are available from the corresponding author on reasonable request.

## Abbreviations

HGI: Hemoglobin glycation index
HbA1c: Glycated hemoglobin
AGEs: Advanced glycation end products
WC: Waist circumference
BMI: Body mass index
BP: Blood pressure
SBP: Systolic BP
DBP: Diastolic BP
FPG: Fasting plasma glucose
TG: Triglycerides
TC: Total cholesterol
ROC: Receiver operating characteristic curve
OR: Odds ratio
RERI: The relative excess risk due to interaction
AP: Attributable proportion due to interaction
SI: The synergy index
HOMA-IR: Homeostasis model assessment of insulin resistance
ACCORD: Action to Control Cardiovascular Risk in Diabetes
CRP: C-reactive protein
